# Medical Society of the County of Erie

**Published:** 1897-08

**Authors:** F. C. Gram


### Society Proceedings.

### MEDICAL SOCIETY OF THE COUNTY OF ERIE.

*Semi-annual Meeting, June 8, 1897.*

REPORTED BY F. C. GRAM, M. D., Secretary.

THE semi-annual meeting of the Medical Society of the County of Erie was held on June 8, 1897, in the rooms of the Buffalo Academy of Medicine, 619 Main street. In the absence of a presiding officer the secretary called the meeting to order shortly after 10 o'clock A. M., and Dr. LUCIEN HOWE was elected temporary chairman.

The minutes of the annual meeting, as well as those of the special meetings held during the past six months, were adopted.

Dr. W. W. POTTER, who was the only one present from the committee on membership, stated that he had not been enabled to inspect the credentials of the applicants for membership to the society, and was therefore not prepared to make any report. Action was deferred until near the close of the meeting, when Dr. Potter stated that he had seen the credentials of Dr. J. Glen Ernest, and recommended his election to membership. The recommendation was unanimously adopted and the applications of the remaining candidates were held over until the next regular meeting.

Applications for membership were received from Drs. Katharine S. Munhall, Henry M. Reinhardt, Wm. Preiss, Renwick R. Ross, D. J. Constantine, Julius Ullman and Edward M. Dooley. Referred to the committee on membership.

Dr. J. B. COAKLEY, chairman of the board of censors, made a verbal report, in which he stated that the attention of the board had been directed to advertising circulars issued by two East Buffalo physicians. They were admonished to desist, which one of them did, while the other left the city.

Their attention also had been called to an alleged female practitioner who had repeatedly been the subject of judicial inquiries. She had treated a lady, and the case terminating fatally her husband had agreed to appear before the grand jury. The district attorney was consulted in regard to the matter and stated that his experience in such cases had not been very encouraging, but promised to look into the matter. The coroner reported to the district attorney that an autopsy revealed the fact that this lady died from fatty degeneration of the heart, and that a chemical examination of the medicine she had used did not reveal any poison. The case was therefore permitted to drop.

The third case which they investigated was that of a person who advertised treatment by electricity. He was informed that he practised in violation of the law and was advised to consult an attorney. He would not promise to desist and the matter was referred to the district attorney. The latter sent the chairman of the board a decision of the supreme court on a similar case, which was adverse to the board. The opinion was written by Justice Daniels, in the case of Smith vs. Lane, "When one may administer treatment to a sick person without a license and is entitled to compensation therefor."—Chapter 436 of 1874—to whom it is not applicable. Hun, 24.

Chapter 436 of 1874, declaring it to be a misdemeanor for any person to practise medicine or surgery who is not authorised so to do by a license or diploma from some chartered school, etc., does not apply to one who undertakes to cure diseases by manipulating the patient's body by rubbing, kneading and pressing it; and such person is entitled to recover a compensation agreed to be paid for such services, although he is not a graduate of a medical school and has no license permitting him to practise either medicine or surgery.

Dr. Coakley quoted the Century dictionary's definition as to the practice of medicine in contradiction to that of the learned justice. He also stated that the district attorney claims his hands are tied and cannot do anything under the present laws, as this decision would be generally accepted.

The report was accepted.

The secretary presented the resignation of Dr. Hopkins as president of the society, which was as follows:

No. 433 Franklin Street,
BUFFALO, January 13, 1897.

To DR. GRAM, Secretary of the Medical Society of the County of Erie.

*Dear Doctor*—Please refer to the by-laws and see who should act as president of the society for the coming year. In case the usual phrase fixing the term of the president's services provides that he shall serve for one year and until his successor is elected and qualified, then I should say the last president, Dr. Thompson, is still in office, otherwise the vice-president elected at the last meeting will have to act as chief. I would advise that you inform the proper person of his responsibility for the coming year, that the business of the society need not suffer. You will please present to the society, when you can, my thanks for the compliment of an election and my sincere regrets at my inability to serve in this important position. Yours,

H. R. HOPKINS.

Dr. Hopkins' resignation was accepted and Dr. Dwyer moved that a committee be appointed to present nominations for the vacancy. The motion was adopted and the chair appointed Drs. Dwyer, Coakley and Brecht.

The secretary also presented the following letter from vice-president Trull:

WILLIAMSVILLE, N. Y., May 26, 1897.

*Dear Dr. F. C. Gram*—Yours of 24th inst. at hand and in reply will say, at the semi-annual meeting on the 8th of June, 1897, I would recommend an election of a vice-president, or in other words, an officer to fill my place, as it may not be possible for me to attend the meeting, and he shall be the presiding officer until the annual meeting, when a full board of presidents will be elected; and I will be excused as a candidate for either office, as I have no ambition for official positions.

Respectfully and sincerely yours,

H. P. TRULL.

This resignation was likewise accepted and the committee on nominations was instructed to nominate a vice-president also.

The committee reported the names of Dr. Lucien Howe, for president, and Dr. J. B. Coakley, for vice-president, to fill the unexpired terms.

The report was adopted and the secretary instructed to cast the ballot of the society for the nominees, which was done.

Dr. EDWARD CLARK stated that Dr. George H. McMichael, who had resigned some time ago, asked for reinstatement.

The president ruled that the application must be made through the membership committee.

Dr. EDWARD CLARK offered the following resolution and moved its adoption:

*Resolved*, That the Medical Society of the County of Erie be reincorporated under the laws of the state of New York, and that the president and secretary of this society are hereby instructed, and given full power, to carry this resolution into effect.

The resolution was adopted.

The secretary presented a letter from Mrs. S. E. Burnett, of Newark, N. J., sister of the late Dr. James B. Samo, in which she thanked Dr. Howe and other members of this society for their kindness to the deceased, as well as for the memorial adopted on his death. She presented the society with Dr. Samo's diploma and certificate. The gift was accepted with thanks.

Dr. Benedict moved the appointment of a committee of three who shall ascertain how many practitioners use and favor the adoption of the metric system. Carried.

The president appointed Drs. Eli Long and Ring a business committee for the session.

Dr. Wm. C. Krauss read a paper on Some facts regarding brain anatomy and brain tumors, and gave a clinical analysis of sixteen cases of brain tumors and exhibited specimens.

Drs. F. Preiss and R. R. Ross led in a discussion on the application of the X-rays in medicine and surgery, exhibiting skiagraphs.

Both papers were discussed by members present.

A motion by Dr. Eli H. Long, calling for the appointment of a business committee to formulate plans for the next regular meeting, was adopted, and the president appointed Drs. E. H. Long, O'Brien and Dunham.

The society then adjourned.

